# A MicroRNA-7 Binding Site Polymorphism in HOXB5 Leads to Differential Gene Expression in Bladder Cancer

**DOI:** 10.1371/journal.pone.0040127

**Published:** 2012-06-29

**Authors:** Junhua Luo, Qingqing Cai, Wei Wang, Hui Huang, Hong Zeng, Wang He, Weixi Deng, Hao Yu, Eddie Chan, Chi-fai NG, Jian Huang, Tianxin Lin

**Affiliations:** 1 Department of Urology, Sun Yat-sen Memorial Hospital, Sun Yat-sen University, Guangzhou, China; 2 Department of Internal Medicine, Cancer Center, Sun Yat-sen University, Guangzhou, China; 3 Department of Urology, Guangzhou General Hospital of Guangzhou Military Command (Guangzhou Liuhuaqiao Hospital), Guangzhou, China; 4 Department of Cardiology, Sun Yat-sen Memorial Hospital, Sun Yat-sen University, Guangzhou, China; 5 Department of Pathology, Sun Yat-sen Memorial Hospital, Sun Yat-sen University, Guangzhou, China; 6 Lin Bai-xin Research Center, Sun Yat-sen Memorial Hospital, Sun Yat-sen University, Guangzhou, China; 7 Division of Urology, Department of Surgery, The Chinese University of Hong Kong, Hong Kong, China; Università di Napoli Federico II, ITALY

## Abstract

**Purpose:**

To investigate the biological function of HOXB5 in human bladder cancer and explore whether the HOXB5 3′-UTR SNP (1010A/G), which is located within the microRNA-7 binding site, was correlated with clinical features of bladder cancer.

**Methods:**

Expression of HOXB5 in 35 human bladder cancer tissues and 8 cell lines were examined using real-time PCR and immunohistochemistry. Next, we explored the biological function of HOXB5 *in vitro* using cell proliferation, migration and colony formation assays. Using bioinformatics, a SNP (1010A/G) was found located within the microRNA-7 binding site in the 3′-UTR of HOXB5. Real-time PCR was used to test HOXB5 expression affected by different alleles. Finally, multivariate logistic regression analysis was used to determine the relationship between SNP (1010A/G) frequency and clinical features in 391 cases.

**Results:**

HOXB5 was frequently over-expressed both in bladder cancer tissues and cell lines. Inhibition of HOXB5 suppressed the oncogenic function of cancer cells. Next, we demonstrated that a SNP (1010A/G), located within the microRNA-7 binding site in the 3′-UTR of HOXB5, could affect HOXB5 expression in bladder cancer mainly by differential binding activity of microRNA-7 and SNP-related mRNA stability. Finally, we also showed the frequency of 1010G genotype was higher in cancer group compared to normal controls and correlated with the risk of high grade and high stage.

**Conclusion:**

HOXB5 is overexpressed in bladder cancer. A miRNA-binding SNP (1010A/G) located within 3′-UTR of HOXB5 is associated with gene expression and may be a promising prognostic factor for bladder cancer.

## Introduction

Urinary bladder cancer is the most common urological tumor in China [1]; however, the mechanisms of bladder cancer tumorigenesis have not been well illustrated. Data show that most of the bladder cancers are induced by carcinogens that damage the DNA. The sensitivity of the transitional epithelium's microenvironment may be another important factor during tumorigenesis [2]. Oncogenes and tumor suppressors have also been reported to play important roles in bladder cancer [3]. Recently, genetic changes including SNPs, deletions, insertions, and changes of DNA copy number have been found to be involved in bladder carcinogenesis.

A homeobox (HOX) is a sequence of about 180 nucleotides within genes that code for a protein domain called homeodomain. Studies showed that HOX genes constitute as much as 0.1–0.2% of the whole vertebrate genome [4]. HOX genes are highly conserved across vast evolutionary distances and encode nuclear proteins that act as transcription factors (TF) during normal organ development [5]. In recent years, the HOX gene family has also been associated with human diseases especially cancers. For instance, loss of HOXA5 in breast cancer [6], over-expression of HOXA10 in acute myeloid leukemia (AML) [7], gene mutations of HOXD4 in renal and colon cancer [8] and overexpression of HOXC4 in human bladder cancer [9] have been reported. HOXB5 (NM_002147.3), located on human chromosome 17, is a member of the HOX gene family that is involved in normal lung and gut development in mouse and human [10]. HOXB5 has been reported to be related to human diseases including AML [11], congenital cystic adenomatoid malformation (CCAM) [12] and bronchopulmonary sequestration (BPS) [13]. Also, the HOXB5 gene was found to be highly expressed in ovarian cancer and was considered to be an important potential targets in the treatment of ovarian cancer [14]. The biological function of HOXB5 in urological carcinomas have not been reported. In a pilot study, we found that HOXB5 was frequently over-expressed in human bladder cancer tissues and cell lines, suggesting that it may be a candidate oncogene in bladder cancer.

In the past ten years, the involvement of microRNAs (miRNAs) in human cancers has been widely studied. MiRNAs repress the expression of the target mRNA by binding to the 3′ untranslated region (3'-UTR) of the mRNA. In human bladder cancer, miRNAs had been shown to be important factors during tumorigenesis. In a previous study, we reported that miRNA-143 and miRNA-125b acted as tumor suppressors in human bladder cancer [15], [16]. In another study it was reported that miR-7 was down-regulated in bladder cancer and may suppress tumor growth by inhibiting growth factor receptor expression and by impairing the anti-apoptotic Akt pathway [17].

Single-nucleotide polymorphisms (SNPs), the most common form of genetic variations in the human genome, contribute to different human phenotypes. SNPs have been associated with many human diseases, especially cancers. In recent years, SNPs located within the miRNA-binding site of a miRNA target (also called miRNA-binding SNP) have been found to be important during tumorigenesis. In a previous study, our group suggested that SNP (1805C/T) in the miR-181a binding site of the Mel-18 gene was related to some clinical features of prostate cancer [18]. In a pilot study, we found a possible miRNA-binding SNP (1010A/G) in the 3′-UTR of the HOXB5 gene using the NCBI SNP database (http://www.ncbi.nlm.nih.gov/snp/) and miRbase (http://www.mirbase.org/).

In this study, we showed that the HOXB5 gene was over-expressed and acted as an oncogene in human bladder cancer. We also found a SNP (1010 A/G) in the 3′-UTR of the HOXB5 gene which is within the miRNA-7 binding site. We have shown that this SNP could affect the expression of HOXB5 mainly by interfering with the function of miRNA-7 and SNP-related mRNA stability; Furthermore, the frequency of 1010G genotype was higher in cancer group compared to normal controls, and was found to be correlated with the risk of high grade and high stage. To our knowledge, this is the first study of the involvement of polymorphisms in the miRNA binding site of HOXB5 in human bladder cancer.

## Materials and Methods

### Patients and Tissues

391 bladder cancer patients were enrolled in our study. This study was approved by the Institute Research Ethics, Sun Yat-sen University, China. Informed consent was written and obtained from all the subjects in our study. All the patients had primary bladder cancers; no previous treatment had been conducted before the operation. The cancer samples were obtained from patients who underwent resection of bladder cancer. The samples were collected between 2007 and 2010 at the Department of Urology, Sun Yat-sen Memorial Hospital, Sun Yat-sen University, Guangzhou, China and Department of Urology, Southwest Hospital, Chongqing, China. All bladder specimens were immediately snap frozen in liquid nitrogen and stored at −80°C. Histology of the tissues was independently evaluated by two pathologists, and the clinical stage of bladder cancer was determined using the 2002 tumor-node-metastasis (TNM) classification system.

### Cell Lines and Cell Culture

Cell lines used in our study were obtained from American Tissue Type Culture Collection (ATCC, Manassas, VA, USA); they include T24, 5637, TCCSUP, HT-1376, UM-UC-3, J82, RT4, EJ and the SV40-transformed kidney cell line 293T. The cells were cultured in a humidified air atmosphere of 5% CO2 at 37°C, and all media were supplemented with 10% fetal bovine serum (Hyclone, Logan, UT, USA). T24 was cultured in McCoy’s 5a medium (modified); 5637 was cultured in RPMI 1640 medium; J82, UM-UC-3, TCCSUP and HT-1376 were cultured in Eagle’s minimum essential medium (EMEM) (Hyclone); and RT4, EJ and SV40-transformed kidney cell line 293T were cultured in Dulbecco’s modified Eagle’s medium (DMEM) (Hyclone).

### RNA Extraction and Quantitative Real-time PCR

Total RNA was extracted from the patients’ bladder samples or cell lines using TRIzol reagent (Invitrogen, Carlsbad, California, USA) according to the manufacturer’s protocol. Quantitative real-time PCR (qPCR) was done using the SYBR green assay (TaKaRa Biotechnology, Dalian, China) on a Roche LightCycler 480 machine (Roche Applied Science, Mannheim, Germany). qPCR was performed as followed: an initial predenaturation step for 30 seconds at 95°C, followed by amplification of 40 cycles at 95°C for 5 seconds and at 60°C for 20 seconds, melting curve analysis was performed at the end. All reactions were done in a 20 µL reaction volume in triplicate. The expression level of HOXB5 was evaluated using the comparative Ct method. GAPDH was used as an internal control. The primers used for HOXB5 were: sense, 5′-TGAAGCACAGGGTTATAACGACCA-3′, antisense, 5′- GCAGCGGGATCCCTGTAAGA-3′; and for GAPDH the primers were: sense, 5′- GAAGGTGAAGGTCGGAGTC-3′, antisense, 5′- GAAGATGGTGATGGGATTTC-3′.

### Immunohistochemistry

Paraffin-embedded, formalin-fixed tissues were cut into 5-µm section, placed on a polylysine-coated slide, deparaffinized in xylene, rehydrated using graded ethanol, quenched for endogenous peroxidase activity in 0.3% hydrogen peroxide and processed for antigen retrieval by microwave heating in 10 mM citrate buffer (pH 6.0). Sections were incubated at 4°C overnight with HOXB5 rabbit polyclonal antibody (1∶100, AbCam, Cambridge, MA, USA). Immunostaining was performed using the ChemMate™ DAKO EnVision™ Detection Kit (DakoCytomation, Glostrup, Denmark), which resulted in a brown precipitate at the antigen site. Subsequently, sections were counterstained with hematoxylin (Zymed Laboratories, South San Francisco, CA, USA) and mounted in nonaqueous mounting medium. The primary antibody was omitted for the negative controls.

### Western Blot

Protein was extracted from bladder cancer tissues and cell lines as described [19]. Briefly, 30 µg of protein from each sample was separated by electrophoresis in a sodium dodecyl sulfate polyacrylamide gel before being transferred to polyvinylidene fluoride membranes (Millipore, Billerica, MA, USA) for 2 hours. Then the membranes were blocked for 1 hour at room temperature using 5% bovine serum albumin (BSA), and incubated in TBST (Tris buffered saline with 0.05% tween) containing rabbit polyclonal IgG2a anti-HOXB5 (1∶1000, AbCam) or GAPDH (1∶1000, Cell Signaling Technology, Beverly, MA, USA) overnight at 4°C. The membranes were incubated with peroxidase-conjugated goat anti-rabbit immunoglobulin (1∶5000, Cell Signaling Technology) as secondary antibody and then visualized using a commercial ECL kit (Pierce, Rockford, IL, USA).

### Cell Transfection with si-HOXB5 and miRNA-7

siRNAs designed to HOXB5 and miRNA-7 mimics were transfected into the bladder cancer cells T24, 5637 and TCCSUP using Lipofectamine-RNAiMAX (Invitrogen). The sequences used for si-HOXB5 were, sense: 5′-GGAUGGACCUCAGCGUCAATT-3′, antisense: 5′-UUGACGCUGAGGUCCAUCCTT-3′; for miR-7 mimics, sense: 5′-CAACAAAUCACAGUCUGCCAUA-3′, antisense: 5′-UAUGGCAGACUGUGAUUUGUUG-3′; and for the negative control, sense: 5′-UUCUCCGAACGUGUCACGUTT-3′, antisense: 5′-ACGUGACACGUUCGGAGAATT-3′. All the RNA oligoribonucleotides were purchased from Genepharma (Shanghai, China). One day before transfection, 2×10^5^ cells were seeded onto a six-well plate. The next day, when the cells reached 70–80% confluence, they were transfected with RNA at a final concentration of 100 nM according to Lipofectamine-RNAiMAX’s protocol. The transfection efficiency measured by qPCR, was 70% for T24, 72% for 5637 and 75% for TCCSUP (data not shown).

### Cell Proliferation Assay

Human bladder cancer cell lines T24, 5637 and TCCSUP were plated onto 6-well plates and incubated at 37°C in a 5% CO2 incubator one day before transfection. After transfection with siRNAs (100 nM) or a negative control for 24 hours, the cells were collected and plated onto 96-well plates for cell viability evaluation using a CCK8 assay (Cell Counting Kit-8) (Dojindo Laboratories, Japan) according to the protocol [20].

### *In vitro* Cell Migration Assay

After the bladder cancer cell lines T24, 5637 and TCCSUP were transfected with si-HOXB5 (100 nM) or nonspecific control (NC) siRNA for 24 hours, the cells were harvested and suspended in 100 µL serum-free medium and then plated (1×10^4^ cells) in the upper compartment of Transwell plates (Corning, NY, USA). The Transwell inserts were then placed into the lower compartment of a 24-well plate containing 600 µL of the medium with 20% FBS as the chemo-attractant. After a 24 hour incubation period, the cells remaining on the top surface of the membrane were removed and the cells on the lower surface were fixed in 100% methanol for 30 minutes, followed by staining with 0.1% crystal violet solution for 30 minutes. Cells that stained purple were defined as positive and the images were captured using a microscope (10×) (Olympus, Center Valley, PA, USA).

### Colony Formation Assay

After transfection with si-HOXB5 (100 nM) or NC siRNA for 24 hours, the human bladder cancer cells T24, 5637 and TCCSUP were collected and placed onto a fresh six-well plate (500 cells for T24, and 1,000 cells for 5637 and TCCSUP). The cells were cultured for about 2 weeks to form colonies. Colonies were fixed with 100% methanol and stained with 0.1% crystal violet in 20% methanol for 15 min. Colony-forming efficiency was calculated as colonies/plated cells ×100%.

### Bioinformatics

The NCBI SNP database (http://www.ncbi.nlm.nih.gov/snp/) was used to find SNPs located within the 3′-UTR of the HOXB5 gene. Four publicly available algorithms, PicTar (http://pictar.mdc-berlin.de/), TargetScan (http://www.targetscan.org/), miRanda (http://www.microrna.org/) and DIANA microT (http://diana.pcbi.upenn.edu/) were used to predict which of the human miRNAs in miRbase (http://www.mirbase.org/) may bind to the 3′-UTR of HOXB5. The miRNAs that were predicted by at least 2 of the algorithms to bind were accepted as candidates for further study. The mRNA secondary structure prediction tool MFOLD (http://mfold.rna.albany.edu/) was used to predict the secondary structure of the HOXB5 mRNA. Small minimal free energy (MFE) indicates high stability of the predicted mRNA secondary structure.

### Luciferase Reporter Assay

We construct luciferase reporter plasmids with the HOXB5 3′-UTR fragment that contained the putative binding sites for the candidate miRNA and subcloned them into the psiCHECK-2 Vector (Promega, Madison, WI, USA) to produce the psiCHECK-2-3′-UTR-WT plasmid. The mutant HOXB5 3′-UTR was generated using the fusion PCR method and then it also subcloned into the psiCHECK-2 Vector to produce the psiCHECK-2-3′-UTR-MUT plasmid. DNA sequencing analysis was used to confirm the sequence of the constructed plasmids.

For the luciferase reporter assay, HEK-293T cells (2×10^4^) were placed onto a 24-well plate one day before transfection. The next day 0.5 µg of either the psiCHECK-2-3′-UTR-WT or the psiCHECK-2-3′-UTR-MUT, and either the miRNA or the negative control were cotransfected into the HEK-293T cells using Lipofectamine2000 (Invitrogen). Assays were performed 48 hours after transfection using the Dual-Luciferase Reporter Assay System (Promega). Luciferase activity was detected using the GloMax-Multi Detection System (Promega). The Renilla luciferase signals were normalized to the internal firefly luciferase transfection control. Transfections were done in triplicate in independent experiments.

### DNA Extraction and HOXB5 Genotyping Analysis

Total DNA was extracted from the patients’ bladder cancer samples and cell lines using QIAamp reagent (QIAGEN, Germantown, MD, USA) according to the manufacturer’s protocol. HOXB5 genotyping was performed using a DNA sequencing assay. A 334 bp DNA fragment containing the SNP in the 3′-UTR of HOXB5 gene was amplified from genomic DNA. The PCR primers used were, forward 5′-GCGCATGAAGTGGAAGAAGG-3′, reverse 5′-TTGGGACAAGCAGAAGGGAG-3′. The amplified DNA fragment was sequenced using GENESCAN software (Applied Biosystems, Foster City, CA, USA).

### Measurement of the Expression of HOXB5 mRNA

The HOXB5 mRNA level was measured in 3 bladder cancer cell lines (5637, J82 and RT4) and 13 bladder cancer tissues. Region-specific Taqman probes were designed to detect the SNP in the 3′-UTR of the HOXB5 mRNA. The cDNA from the cell lines and cancer tissues were subjected to qPCR and the fluorescence (VIC for 1010A, FAM for 1010G) was measured using LightCycler 480 Probes Master (Roche Applied Science, Mannheim, Germany).

Genomic DNA was also extracted from cell lines and cancer tissues as mentioned. As an internal control, qPCR was performed to determine the genomic DNA levels of HOXB5 using the same region-specific Taqman probes.

### HOXB5 mRNA Half-life

qPCR was also used to measure the half-life of the HOXB5 mRNA. 1×10^6^ T24 and TCCSUP bladder cancer cells were plated onto a 10-cm dish one day before actinomycin D (5 µg/ml), which inhibits genetic transcription, was added to the cells. After treated with actinomycin D, the cells were lysed using TRIzol at different time points, 0 h, 4 h, 8 h, 12 h, 24 h and 48 h. Total RNA was extracted and the HOXB5 mRNA level was quantified by qPCR using the Taqman assay as previously described above.

### Statistic

All data are expressed as the mean ± SEM from at least three separate experiments. The differences between groups were analyzed using Student’s t test when only two groups were compared, or, by one-way analysis of variance (ANOVA) when more than two groups were compared. The age-adjusted odds ratio (aOR) and 95% confidence interval (CI) for the relationship between the HOXB5 3′-UTR genotype frequencies and clinical or histological features were determined by multivariate logistic regression analysis using SPSS 17.0 with age considered as a factor. All statistical tests were two-sided. Differences were considered statistically significant at p<0.05.

## Results

### HOXB5 was Over-expressed in Human Bladder Cancer Tissues and Cell Lines

**Figure 1:**
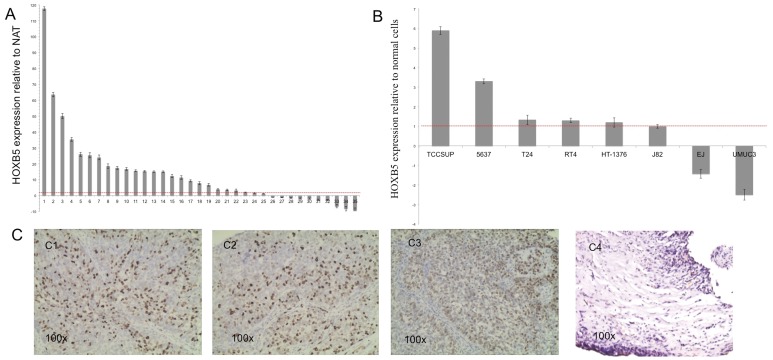
HOXB5 is over-expressed in human bladder tumors. A. Expression of HOXB5 in 35 bladder cancer tissues relative to normal adjacent tissues (NAT). Columns above the X-axis indicate overexpression of HOXB5; those below the X-axis indicate down-expression of HOXB5 relative to NAT. B. Expression of HOXB5 in eight bladder cancer cell lines relative to normal bladder cells. Columns above the X-axis indicate overexpression of HOXB5; those below the X-axis indicate down-expression of HOXB5 relative to normal cells. Fold changes >1 was considered to be positive. C. HOXB5 expression in primary transitional cell bladder cancer tissues detected by immunohistochemistry. C1 and C2, Bladder cancer tissues, G2 grade. C3, Bladder cancer tissue, G3 grade. C4, Normal bladder tissue. All images are ×100. Staining: brown, HOXB5.

RNA was extracted from 35 bladder cancer patients and 8 bladder cancer cell lines and the expression of HOXB5 was measured using qPCR. As shown in Figure 1A, of 35 samples, 23 (∼70%) exhibited higher expression of HOXB5 compared with normal adjacent tissue (NAT). The expression of HOXB5 was also higher in 6 of 8 bladder cancer cell lines (TCCSUP, 5637, T24, RT4, HT-1376, and J82) than in normal bladder cells (Figure 1B). Immunohistochemical studies using the HOXB5-specific antibody confirmed that the expression of HOXB5 is higher in bladder cancer tissues than normal bladder tissues (Figure 1C). However, there was no correlation between the expression of HOXB5 and the tumor grade or stage (data not shown). These results suggested that the overexpression of HOXB5 may be common in some bladder cancer tissues and in cell lines.

### HOXB5 Promotes Cell Proliferation and Migration of Bladder Cancer Cells

**Figure 2:**
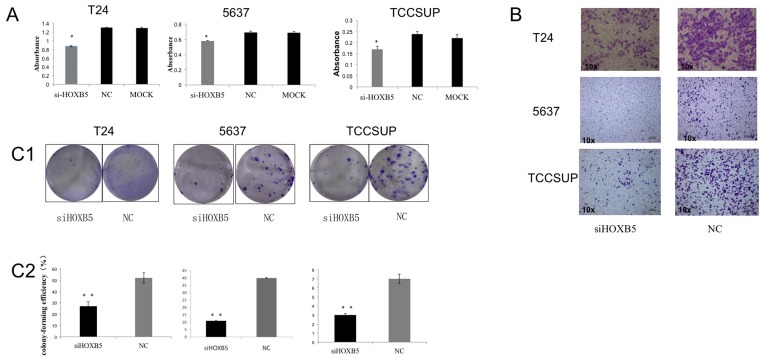
si-HOXB5 inhibited the biological function of bladder cancer cells *in vitro*. A. Proliferation of bladder cancer cell lines T24, 5637 and TCCSUP. A CCK8 assay was used to examine cell growth of bladder cancer cells. B. Migration of bladder cancer cell lines. Left column, si-HOXB5 transfected group; right column, NC siRNA transfected group. All images are ×10. Staining: purple, migration cells. C. Colony formation (C1) and colony-forming efficiency (C2) of bladder cancer cells after transfection with si-HOXB5 or NC siRNA. Colony-forming efficiency  =  colonies/plated cells ×100%. *p<0.05, **p<0.01. NC, nonspecific control. MOCK, Lipofectamine only.

We found that HOXB5 was over-expressed in bladder cancer tissues and in cell lines, indicating that HOXB5 may act as an oncogene. To investigate the oncogenic function of HOXB5, we transfected si-HOXB5 and NC siRNA into T24, 5637 and TCCSUP cells. 48 hours after transfection, a CCK8 assay showed that cell growth was significantly decreased in si-HOXB5 transfected groups compared with the NC group or mock group (Lipofectamine only) (Figure 2A, p<0.05). We also found that the migration ability of si-HOXB5 transfected cells was significantly inhibited compared with the NC group or mock group (Figure 2B). These results indicated that HOXB5 may promote cell proliferation and the migration of bladder cancer cells, consistent with a role of an oncogene.

### si-HOXB5 Suppresses Clonogenicity *in vitro*

To further explore the potential role of HOXB5 in tumorigenesis, we investigated the effect of HOXB5 on colony formation of cancer cells *in vitro*. Three bladder cancer cell lines (T24, 5637 and TCCSUP) were transfected with an si-HOXB5 or NC duplex, and allowed to grow at very low density (500 cells for T24, 1,000 cells for 5637 and TCCSUP) for about 14 days. Notably, si-HOXB5 inhibited, both in size and number, the ability of bladder cancer cells to form colonies (Figure 2 C1). Further, the si-HOXB5 transfected cells showed lower colony-forming efficiency than the NC-transfected cells (Figure 2 C2, p<0.01). These data further supported the oncogenic effect of HOXB5 in bladder cancer cells.

### SNP-1010A/G is Located within miRNA-7 Binding Site in HOXB5 3′-UTR

**Figure 3:**
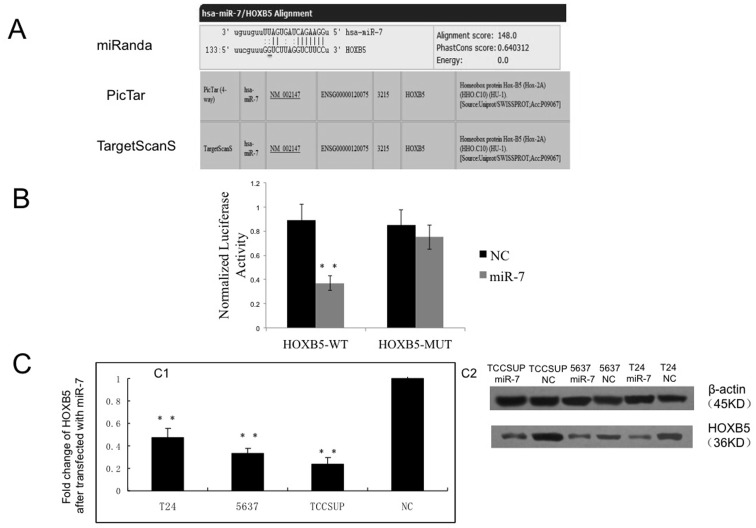
HOXB5 is a target of miR-7. A. HOXB5 was predicted as a direct target of miR-7 by miRanda, PicTar and TargetScan. B. Luciferase analysis in HEK-293T cells. WT, wild type. MUT, mutant type. C. Effect of miR-7 overexpression on the expression levels of endogenous HOXB5 in T24, 5637 and TCCSUP cells. Endogenous HOXB5 mRNA and protein levels were assayed by qPCR (C1) and Western blot (C2) respectively. β-actin, internal control. **p<0.01, compared with NC transfectants. NC, nonspecific control.

We found SNP rs9299 (1010 A/G) is located within the 3′-UTR of the HOXB5 gene using the NCBI SNP database. HOXB5 was also predicted to be one of the target genes of miRNA-7 according to 3 of the different systemic bioinformatics software that we used, and the SNP (1010 A/G) was located within the miRNA-7 binding site (Figure 3A).

To validate HOXB5 as a bona fide target of miR-7, a human HOXB5 3′-UTR fragment containing either the wild-type or mutant miR-7-binding sequence was subcloned downstream of the Renilla luciferase reporter gene as described in the Materials and Methods section. The relative luciferase activity of the reporter containing the wild-type HOXB5 3′-UTR was significantly suppressed when miR-7 was co-transfected (p<0.01). In contrast, the luciferase activity of the reporter containing the mutant miR-7-binding site was almost unaffected (p>0.05) (Figure 3B).

To further explore the regulation of HOXB5 expression by miR-7, we transfected miR-7 mimics and NC into the cell lines T24, 5637 and TCCSUP. After 48 hours, we examined the HOXB5 mRNA and protein levels using qPCR and western blot. We found that the HOXB5 mRNA and protein levels were down-regulated in the miR-7 transfected groups compared with the NC groups (Figure 3 C1 & C2).

These results indicated that miR-7 may regulate HOXB5 expression at both the post-transcription and mRNA levels.

### SNP 1010A/G affects HOXB5 Expression

**Figure 4:**
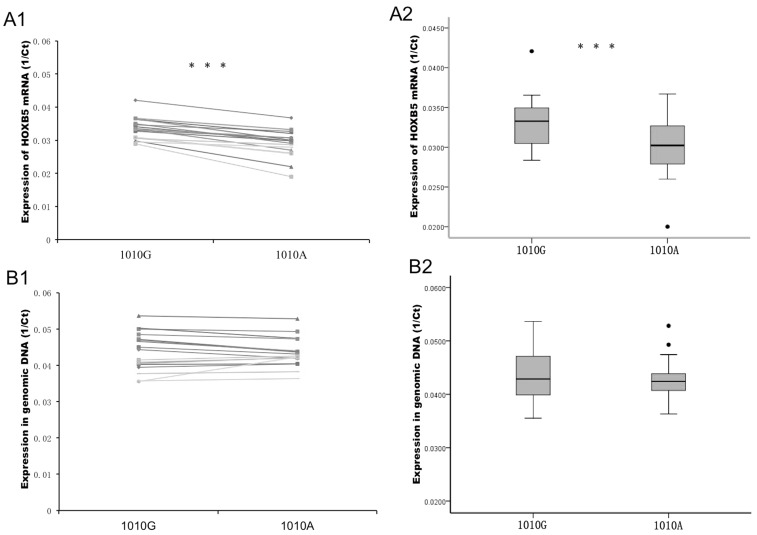
Expression of HOXB5 mRNA for each allele in heterozygous bladder cancer tissues and cell lines. A1. Expression of mRNA for each allele in the heterozygous GA genotype cell lines (5637, RT4 and J82) and tissues (13 cases). A2. Expression of mRNA (Mean) for each allele in heterozygous GA genotype cell lines and tissues. Y-axis, expression of HOXB5 mRNA. Ct: cycle threshold, calculated from Realtime-PCR machine. B1. Expression of the heterozygous genomic DNA as an internal control. B2. Expression of the heterozygous genomic DNA (Mean) as an internal control. Y-axis, expression in genomic DNA. ***p<0.001.

To investigate the affect of SNP 1010A/G on the expression of HOXB5, we examined the mRNA levels of HOXB5 for the 1010A and 1010G alleles in the heterozygous GA genotype bladder cancer tissues (13 cases) and cell lines (5637, RT4 and J82), using the Taqman assay as described above. We found that the expression of the HOXB5 mRNA with the 1010G allele was significantly higher than the mRNA with the 1010A allele in both cancer tissues and cell lines (Figure 4A1 and A2). However, the expression ratio of 1010G to 1010A in the genomic DNA from these heterozygous cancer tissues and cell lines were similar (Figure 4B1 and B2). These results showed that the 1010A/G SNP in the HOXB5 3′-UTR affected the expression of HOXB5 mRNA.

### The Different Alleles Affect mRNA Stability and HOXB5 Expression Levels

**Figure 5:**
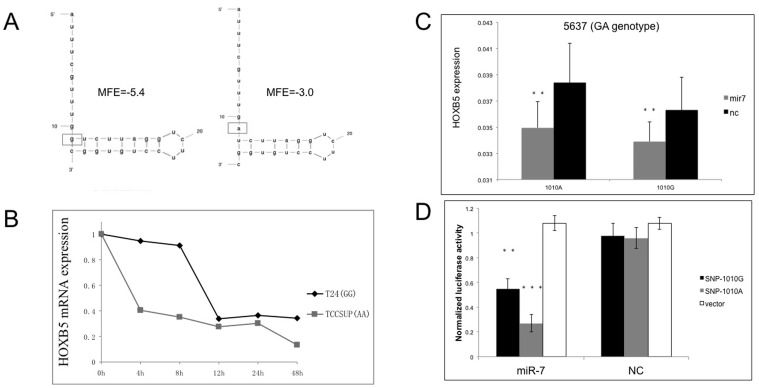
Different alleles affect HOXB5 mRNA stability and the activity of miR-7 binding. A. Secondary structures of HOXB5 mRNA predicted by MFOLD. Minimal free energy (MFE) may reflect mRNA stability. B. Half-life of HOXB5 mRNA in T24 (GG genotype) and TCCSUP (AA genotype) cells. The half-life for the mRNA with the G allele was about 11 hours, and about 3.7 hours with the A allele. C. HOXB5 expression level after transfection with miR-7 relative to NC in 5637 cells (GA genotype). Both A and G alleles of the mRNA transfected with miR-7 exhibited down-regulation relative to the NC group. The level of HOXB5 mRNA with the A allele decreased more than mRNA with the G allele. D. Luciferase analysis in HEK-293T cells of miR-7 activity. Vector, psiCHECK-2 Vector. *p<0.05, **p<0.01, ***p<0.001.

To predict possible mechanisms how the 1010A/G SNP results in differential HOXB5 expression levels, we used the mRNA secondary structure prediction tool MFOLD to predict the secondary structure of the mRNAs with the A and G alleles. We found that the predicted minimal free energy (MFE) of the secondary structure of the mRNA with the G allele was lower than that of the mRNA with the A allele (−5.4 vs −3.0). This result indicated that the structure of the mRNA with the G allele may be more stable than that for the mRNA with the A allele (Figure 5A).

To further explore which allele (A or G) conferred more stability, we measured the mRNA half-life because it has been shown that the steady state of mRNA is closely related to the mRNA half-life [21]. We examined the half-life of HOXB5 mRNA in the homozygous T24 and TCCSUP bladder cancer cells (GG for T24 and AA for TCCSUP) after treatment with actinomycin D, using qPCR. The results showed that the half-life of HOXB5 mRNA in the cells with the GG genotype was 3.5 fold (11 h) than the mRNA half-life (3.4 h) in the cells with the AA genotype (Figure 5B), indicating that the mRNA with the G allele was more stable than the mRNA with the A allele. This different stability on the two mRNAs may be the possible mechanism that explains the different effect of the SNP on HOXB5 expression.

### The Binding Activity of miR-7 for Different Alleles of mRNA affects HOXB5 Expression Level

We transfected miR-7 to a bladder cancer cell line (5637) with the heterozygous GA genotype for 48 hours and measured the HOXB5 mRNA level using the Taqman assay. We observed that the overexpression of miR-7 could significantly inhibit the expression level of HOXB5 mRNA compared with the NC group? Interestingly, the expression level of the HOXB5 mRNA with the A allele decreased much more than the level of the mRNA with the G allele (Figure 5C), indicating that the binding of miR-7 to the HOXB5 mRNA with the A allele was greater than the mRNA with the G allele.

To validate our hypothesis, we carried out a luciferase assay. The relative luciferase activity was suppressed much more in the reporter containing the 1010A transfected with miR-7 than that containing the 1010G allele (Figure 5D). These results showed that the binding activity of miR-7 with either the 1010A or 1010G allele may be another important mechanism involved in the different HOXB5 expression levels affected by the SNP.

### The Association between the 1010A/G HOXB5 Genotype Frequency and Bladder Cancer

Next, we examined the association between 1010A/G HOXB5 genotype frequency and the clinical features of bladder cancer. DNA was extracted from 391 patients with bladder cancer that was confirmed by pathologists, and from 391 normal controls, and the SNP (1010A/G) genotypes for each sample were analyzed. We found that G allele (AG+GG) genotypes were associated with the risk of high grade (Grade 2 and 3, aOR = 4.25, p<0.001, Table 1) and high stage (T2–T4, muscle invasive type, aOR = 2.25, p = 0.003, Table 2) cancers as against low grade (Grade1) and low stage (T1, non-muscle invasive type) cancers. We also showed that the frequency of G genotypes (AG+GG) was higher in bladder cancer group compared with the normal controls (aOR = 1.48, p = 0.017) (Table 3).

**Table 1:** Genotype frequencies of the HOXB5 polymorphism in bladder cancer subgroups (G1 and G2–G3 groups).

HOXB5 1010A/G genotype	G1	G2–G3	aOR^a^ (95%CI^b^)	p
	N^c^ (%)	N (%)		
AA	51 (37.8%)	32 (12.5%)	Ref	
AG	68 (50.4%)	163 (63.7%)	3.82 (2.26–6.48)	0.001
GG	16 (11.9%)	61 (23.8%)	6.07 (2.99–12.31)	0.001
AG+GG (against AA)	84 (62.2%)	224 (87.5%)	4.25 (2.58–7.07)	***<***0.001
AG+AA (against GG)	119 (88.1%)	195 (76.2%)	0.40 (0.22–0.73)	0.003

a age-adjusted odds ratio,

b 95% confidence interval,

c Numbers of people.

**Table 2:** Genotype frequencies of the HOXB5 polymorphism in bladder cancer subgroups (Non-muscle invasive and Muscle-invasive groups).

HOXB5 1010A/G genotype	Non-muscle invasive	Muscle-invasive	aOR^a^ (95%CI^b^)	p
	N^c^ (%)	N (%)		
AA	59 (26.8%)	24 (14%)	Ref	
AG	119 (54.1%)	112 (65.5%)	2.31 (1.35–3.47)	0.002
GG	42 (19.1%)	35 (20.5%)	2.05 (1.06–3.94)	0.031
AG+GG (against AA)	161 (73.2%)	147 (85.9%)	2.25 (1.33–3.79)	0.003
AG+AA (against GG)	178 (80.9%)	136 (79.5%)	0.91 (0.56–1.51)	0.917

a age-adjusted odds ratio,

b 95% confidence interval,

c Numbers of people.

**Table 3:** Genotype frequencies of the HOXB5 polymorphism in controls and bladder cancer groups.

HOXB5 1010A/G genotype	Controls	Bladder cancer	aOR^a^ (95%CI^b^)	p
	N^c^ (%)	N (%)		
AA	113 (28.6%)	83 (21.2%)	Ref	
AG	195 (50.4%)	231 (59.1%)	1.58 (1.12–2.22)	0.009
GG	83 (21%)	77 (19.7%)	1.263 (0.83–1.92)	0.276
AG+GG (against AA)	278 (71.4%)	308 (78.8%)	1.487 (1.07–2.06)	0.017
AG+AA (against GG)	308 (79%)	314 (80.3%)	0.922 (0.65–1.31)	0.646

a age-adjusted odds ratio,

b 95% confidence interval,

c Numbers of people.

## Discussion

The HOX gene family has recently been identified as one of the main factors in the normal development of the human organs. The HOXB5 gene, which was found to be involved in lung and gut development, was reported to be an important factor in human disease, including cancers [14]. Here, we showed that the HOXB5 gene was frequently over-expressed in human bladder cancer tissues and in cancer cell lines. *In vitro* experiments showed that HOXB5 may act as an oncogene in human bladder cancer. We found a SNP (1010 A/G) in the 3′-UTR of the HOXB5 gene, which was also within a miRNA-7 binding site. We observed that this SNP could affect the expression of the HOXB5 gene. Accordingly, we proposed that miR-7 binding activity and mRNA stability which can be affected by SNP may be involved in the differential expression of HOXB5. Finally, the frequency of 1010G genotype was higher in bladder cancer group compared to normal controls, and was related to the risk of high grade and high stage bladder cancers.

Homeobox genes code for transcription factors that are primarily involved in embryonic development. Several homeobox gene families, including HOX, EMX, PAX, MSX and many isolated divergent homeobox genes have been identified. The HOX gene family is the one that has most often been found to play a role in regulating network structure organization [22]. Over 10 years ago, the HOX genes were found to control embryonic organ-specific patterning. During embryogenesis, these genes were shown to code for transcription factors that regulate the expression of subordinate genes [23]. The HOX gene family was also found to be involved in human tumorigenesis [6], [7], [8], [9]. Among HOX gene family, HOXB5 gene has been found to play a role in the patterning of airway branches during mouse lung morphogenesis in Volpe et al’s study [10]. Later, they found HOXB5 gene was also related to human lung morphogenesis and may play a role in controlling airway patterning [19]. The HOXB5 gene was found to related with vasculogenesis by its interaction with vascular endothelial growth factor receptor-2 (VEGFR-2) and angiopoietin-2 (Ang2) [24], [25], indicating that HOXB5 may be involved in tumorigenesis. Until now, the biological function of HOXB5 in human bladder cancer has not been reported. In the present study, we found that HOXB5 was over-expressed in human bladder cancer and our *in vitro* experiment showed that HOXB5 may act as an oncogene in bladder cancer.

In recent years, genome-wide association studies (GWAS) have given us a deeper insight into the mechanisms related to genomic changes in various cancers. Chang *et al.* identified several susceptibility loci in human bladder cancer, including rs9642880 (nearest gene: MYC), rs710521 (nearest gene: TP63), and rs2294008 (nearest gene: PSCA) among others [26].

The involvement of miRNAs in human cancer has been discovered recently. In a previous study, we reported that miRNA-143 and miRNA-125b act as tumor suppressors in human bladder cancer by binding to the oncogenes RAS and E2F3 respectively [15], [16]. 3′-UTR polymorphisms in certain genes have been reported to related with human disease, including hereditary thrombophilia [27], urolithiasis [28], and increased sensitivity to 5-fluorouracil chemotherapy [29]. SNPs in miRNA-binding sites have recently been discovered. Yu et al. conducted a genome-wide analysis of SNPs located in the miRNA-binding sites of the 3′-UTR of various human genes associated with human cancers. They found 1,265 SNPs that were located within the miRNA-binding sites, and suggested that these SNPs may affect expression of the miRNA binding target [30]. Mishra et al. showed that SNP 829C/T located within the miRNA-24 binding site of the 3′-UTR of the DHFR gene led to overexpression of its target gene and resulted in resistance to methotrexate [21]. In a previous study from our group, we reported that the Mel-18 gene functioned as a tumor suppressor in prostate cancer, and a SNP (1805A/G) in the miRNA-181a binding site correlated with Mel-18 expression and clinical features in prostate cancer [18].

Until now, no SNPs in miRNA-binding sites have been reported in human bladder cancer. MiR-7 was shown to be down-regulated in human glioblastoma and bladder cancer and a further study showed that miR-7 may suppress tumor growth in human bladder cancer by inhibiting growth factor receptor expression and by impairing the antiapoptotic Akt-pathway [17]. Bioinformatics analyses predicted a miR-7-binding SNP (1010A/G) within the 3′-UTR of the HOXB5 gene. Here, we reported a SNP (1010A/G) that was located within the miR-7 binding site of the 3′-UTR of the HOXB5 gene, and found that the different SNP (A or G) genotype could affect HOXB5 mRNA expression. Many 3′-UTR polymorphisms had been shown related with altered gene expression, but the possible mechanisms were not fully understood [21]. We propose that the SNP (1010A/G) may affect the expression of HOXB5 in bladder cancer by differential mRNA stability and binding activity of miR-7. Furthermore, multivariate logistic regression analysis showed that genotypes with the G allele (GG and AG) were associated with the risk of high grade (Grade 2 and 3, aOR = 4.25, p<0.001) and high stage (T2–T4, muscle invasive type, aOR = 2.25, p = 0.003) cancers. We also showed that compared with normal controls, the genotypes with the G allele were associated with the risk of bladder cancer (aOR = 1.48, p = 0.017). These results suggested that the SNP located within the miR-7 binding sites may affect HOXB5 expression, which in turn may affect bladder tumorigenesis. In addition, this SNP may have the potential to become a prognostic factor for bladder cancer.

miRNA binding site polymorphisms have only recently been investigated. These polymorphisms may not only affect gene expression, but could also have a relationship with clinical features of cancer or even with the prognosis of cancer. In future studies, we intend to source many more bladder cancer cases and use them to carry out a longer-term study to discover whether or not this SNP (rs9299) could be a good prognostic and prognosis factor for bladder cancer.

In summary, in this study we showed for the first time that the HOXB5 gene may act as an oncogene in human bladder cancer. We found that a SNP (1010A/G) within the miR-7 binding site of HOXB5 3′-UTR affects HOXB5 expression and this SNP may be correlated with bladder tumorigenesis and the risk of high grade and high stage human bladder cancers. These results suggested a possible mechanism for the effects of the miRNA binding site polymorphism during bladder tumorigenesis and revealed a possible prognostic and prognosis factor for bladder cancer.
